# Internalizing and externalizing traits predict changes in sleep efficiency in emerging adulthood: an actigraphy study

**DOI:** 10.3389/fpsyg.2015.01495

**Published:** 2015-10-06

**Authors:** Ashley C. Yaugher, Gerianne M. Alexander

**Affiliations:** Department of Psychology, Texas A&M UniversityCollege Station, TX, USA

**Keywords:** actigraphy, sleep, emerging adult, internalizing disorders, externalizing disorders, impulsivity, psychopathology, antisocial personality

## Abstract

Research on psychopathology and experimental studies of sleep restriction support a relationship between sleep disruption and both internalizing and externalizing disorders. The objective of the current study was to extend this research by examining sleep, impulsivity, antisocial personality traits, and internalizing traits in a university sample. Three hundred and eighty six individuals (161 males) between the ages of 18 and 27 years (*M* = 18.59, *SD* = 0.98) wore actigraphs for 7 days and completed established measures of disorder-linked personality traits and sleep quality (i.e., Personality Assessment Inventory (PAI), Triarchic Psychopathy Measure, Barratt Impulsiveness Scale-11, and the Pittsburgh Sleep Quality Index). As expected, sleep measures and questionnaire scores fell within the normal range of values and sex differences in sleep and personality were consistent with previous research results. Similar to findings in predominantly male forensic psychiatric settings, higher levels of impulsivity predicted poorer subjective sleep quality in both women and men. Consistent with well-established associations between depression and sleep, higher levels of depression in both sexes predicted poorer subjective sleep quality. Bidirectional analyses showed that better sleep efficiency decreases depression. Finally, moderation analyses showed that gender does have a primary role in sleep efficiency and marginal effects were found. The observed relations between sleep and personality traits in a typical university sample add to converging evidence of the relationship between sleep and psychopathology and may inform our understanding of the development of psychopathology in young adulthood.

## Introduction

The co-occurrence of sleep problems and affective symptoms such as anxiety and depression is well established (Lavigne et al., 1999; Alfano et al., 2007, 2009). In addition, emerging research indicates sleep deficiency increases externalizing, antisocial behaviors such as aggression and conduct problems (Kamphuis et al., 2012). For example, sleep disordered breathing in children and adolescents is associated with parental reports of increased aggression, hyperactivity, and oppositional behaviors (Chervin et al., 2003; Rosen et al., 2004; Perfect et al., 2013). Similar associations between self-reported sleep problems, aggression and delinquent acts are also found in studies of juvenile offenders (Ireland and Culpin, 2006; Clinkinbeard et al., 2011; Sarchiapone et al., 2014). The possibility that a causal relationship between sleep problems and externalizing behavior may underlie these general findings is suggested by experimental research showing total or partial sleep deprivation in healthy individuals produces greater emotional dysregulation, impulsivity, aggression and oppositional behaviors (Dinges et al., 1997; Talbot et al., 2010; Anderson and Platten, 2011; McGlinchey et al., 2011; Dagys et al., 2012; Baum et al., 2014; Rossa et al., 2014).

It has also been well established that externalizing (e.g., impulsivity, hyperactivity) and internalizing (e.g., loneliness, depression, anxiety) symptoms increase sleep difficulties (Cousins et al., 2011; Doane and Thurston, 2014). For example, studies of non-medicated adults diagnosed with Attention Deficit Hyperactivity Disorder (ADHD), a disorder with hyperactivity, inattention, and impulsivity as main components, report higher incidence of sleep problems on self-report measures of sleep quality when they are more hyperactive and impulsive (Mahajan et al., 2009). Studies in adolescence have found increased incidence of self-reported loneliness and stress results in decreased sleep duration and other sleep problems (Doane and Thurston, 2014). This same study showed that sleep difficulties also resulted in increases in reported stress and loneliness (Doane and Thurston, 2014). These results suggest that there is a bidirectional relationship between sleep problems and psychopathology. In other words, the relationship between sleep-related problems and internalizing/externalizing symptomatology can both be causal as well as co-occur with one another. However, studies of this type are often limited by number of days as well as relatively low level of participants (Doane and Thurston, 2014). For example, in a longitudinal study of 82 adolescents they found a bidirectional relationship between objective sleep measures and internalizing symptoms, but only three nights of objective sleep data was required (Doane and Thurston, 2014).

Increased aggression and impulsivity associated with sleep restriction is suggestive of mild prefrontal lobe dysfunction (Killgore et al., 2008). The hypothesis that sleep restriction alters prefrontal lobe functioning is supported by fMRI research showing 35 h of sleep deficiency in healthy adults results in a hyper-limbic response by the amygdala to negative visual stimuli as a failure of prefrontal cortical control (Yoo et al., 2007). In a separate report (Mullin et al., 2013), a day of total sleep deprivation in emerging adults decreased activation in areas associated with social cognition (i.e., the medial prefrontal cortex) and increased activation in reward systems (i.e., the ventral striatum) during the winning of a monetary reward. In addition, shorter typical sleep durations (as measured by actigraphy in the 5-days prior to the period of sleep deprivation) were associated with increased activation in the medial prefrontal default-mode network during reward-related decisions, a response that is contrary to findings that this neural network is typically deactivated during cognitive tasks (Mullin et al., 2013). Given this pattern of brain response to sleep deprivation, others have suggested that the continued maturation of the prefrontal-subcortical circuits central to emotional control in emerging adulthood (Giedd, 2004) coupled with lifestyles that often compromise optimal sleep schedules (Carskadon et al., 1998; Dahl and Lewin, 2002; Jenni et al., 2005) may explain in part the increased risk-taking behaviors, as well as the emotional and behavioral dysregulation associated with this developmental period (Dahl, 1996; Casey et al., 2008; Casey and Caudle, 2013).

Chronic sleep loss in adolescence and young adulthood may also result in long-term malfunctioning of prefrontal-subcortical circuits regulating emotion (Beebe, 2011), particularly in combination with episodes of acute sleep loss (Mullin et al., 2013; Rossa et al., 2014). The occurrence of sleep problems in individuals with externalizing personality disorders appears consistent with this possibility. For example, high impulsivity and high criminality in disorders associated with prefrontal lobe abnormalities (Helpern et al., 2011; Sundram et al., 2012) [i.e., Antisocial Personality Disorder (APD), Borderline Personality Disorder (BPD), and ADHD] are associated with less sleep satisfaction, more sleep-wakefulness and daytime dysfunction, higher levels of slow wave sleep (SWS), lower levels of sleep efficiency and overall sleep duration, as measured by actigraphy and polysomnography (Lindberg et al., 2003; Tworoger et al., 2005; Boonstra et al., 2007). Similarly, in studies of patients in forensic psychiatric settings, individuals reporting sleep problems score higher on measures of antisocial personality traits (Kamphuis et al., 2013) and subjective and objective measures of aggression (Kamphuis et al., 2014).

Personality characteristics (i.e., externalizing and internalizing traits) exist on a continuum that begin to develop in adolescence when sleep is an important factor in developing psychopathology (Ireland and Culpin, 2006; Clinkinbeard et al., 2011). Consistent with a dimensional view of behavior (Rivas-Vazquez et al., 2004; Krueger et al., 2005; Dedovic et al., 2014), sleep problems and externalizing traits exist on a continuum from mild levels in subclinical populations to severe levels in clinical disorders suggesting it may be useful in understanding the role of sleep disruption in the development of personality disorders to first establish whether sleep and externalizing personality traits covary along a continuum of typical behavior. Some evidence supports this possibility. Older, healthy adults reporting longer sleep durations, for example, have greater functional connectivity between the prefrontal cortex and score lower on a measure of antisocial personality traits (Killgore et al., 2013). Similarly, in a large self-report survey of adolescents (*n* = 11, 788) across 11 countries, sleep hours per night correlated with risk for suicidal ideation, conduct, and peer problems (Sarchiapone et al., 2014). In research using data from Wave one of the National Longitudinal Study of Adolescent Health (Add Health) groups reporting seven or fewer and five or fewer also reported greater property delinquency and violent delinquency respectively (Clinkinbeard et al., 2011). However, in contrast to studies suggesting modest sleep deficits in younger individuals increase antisocial behavior, in research using data from the Youth Risk Behavior Survey (*n* = 15, 364) sleep deficits above 5 h were largely unrelated to negative outcomes (Meldrum and Restivo, 2014). Thus, evidence of a linear relationship between sleep and externalizing problems in normal populations is equivocal.

The primary aim of the current research was to examine the nature of the relationship between antisocial personality traits in emerging adulthood using an objective measure of sleep and wakefulness and validated self-report measures of impulsivity and psychopathy, two traits strongly associated with APD (Harty et al., 2010). Although others have examined related behaviors such as delinquency, conduct problems, and aggression, to our knowledge this is the first examination of sleep and a global measure of psychopathic traits. Further, given evidence that men may be more vulnerable to the effects of sleep restrictions on externalizing behavior (Acheson et al., 2007) and a greater male prevalence of externalizing disorders such as APD and ADHD (Kessler et al., 2005), we also examined whether a relationship between externalizing behavior and sleep disturbance may vary according to gender. Finally, as others have found a relationship between sleep duration and other maladaptive personality traits (i.e., stable personality characteristics over time) in relatively small samples of adults (< 50) (Kahn-Greene et al., 2007; Tkachenko et al., 2014), we included scales from the Personality Assessment Inventory (PAI) in a second set of questionnaires administered to a subset of participants following sleep data collection.

We hypothesized that as externalizing traits (i.e., trait impulsivity, antisocial traits, and psychopathic traits) increased levels of sleep satisfaction, efficiency (i.e., percent sleep), and duration (i.e., sleep time in minutes) would decrease. We also hypothesized that men would report higher rates of externalizing traits than women and that this higher report of externalizing traits would result in poorer sleep efficiency, duration, and satisfaction. We hypothesized that increased internalizing and externalizing personality traits (i.e., traits of depression, borderline, antisocial, and anxiety) would result in sleep disturbances. Finally, we hypothesized that decreases in sleep efficiency and sleep duration would result in increased externalizing and internalizing traits (i.e., the bidirectional relationship between sleep and psychopathology in a large sample).

## Materials and methods

### Participants

Four hundred and two undergraduate students completed the experiment, as partial fulfillment of course requirements, a protocol that was approved by the Institutional Review Board. Sample size was determined following power recommendations by Cohen (Cohen, 1992) with alpha set at 0.05, and effect size estimates were based on meta-analytic findings showing a medium effect size for the relationship of psychopathy and risk assessment (Dolan and Doyle, 2000), and a meta-analysis of children with impulsive traits (i.e., ADHD) and the relationship to sleep disturbance (limb movement) showing a smaller effect size (*d* = 0.26) (Sadeh et al., 2006). Actigraph data was inspected to ensure that participants wore the watch for at least 5 days during the seven day protocol and were excluded in analyses if they did not meet the criteria. Sleep data was unavailable for 16 participants for reasons including a failure to wear the actiwatch and battery malfunction. After exclusions, a total of 386 participants (age range 18–27, *M* = 18.59, 161 males) were included in the study.

### Measures

#### Sleep measures

Both subjective and objective measures of sleep satisfaction and quality were used in the current study to examine sleep efficiency (i.e., percent sleep), duration (i.e., sleep time in minutes), and self-reported sleep satisfaction (i.e., PSQI).

##### PSQI

The Pittsburgh Sleep Quality Index (PSQI), a widely-used 19 item self-report questionnaire measured sleep quality and disturbances in the past month (Buysse et al., 1989). The current study found similar Cronbach's alpha scores that have been reported in non-clinical samples (α = 0.74) (Mollayeva et al., 2015). Furthermore, a recent meta-analysis has confirmed that the PSQI is a reliable and valid measure of sleep quality and continues to be widely used in both clinical and non-clinical settings (Mollayeva et al., 2015).

##### Accelerometer

A small accelerometer (Actiwatch, AW64, Philips Respironics) provided objective measures of sleep and wakefulness. Consistent with previous research methods (Ruiz et al., 2011), in the current study, accelerometers were initialized to capture movement counts within 15 s intervals and worn for 7 days. Dependent variables were average sleep duration (i.e., sleep time in minutes) and average sleep efficiency (i.e., percent sleep) throughout the week, as these variables are often used and are highly correlated with other objective measures of sleep (e.g., polysomnography) (Kaplan et al., 2012). Sleep Efficiency is calculated by the accelerometer and indicates how efficiently each participant slept (i.e., the percentage of uninterrupted sleep beginning at sleep onset and ending at wakefulness; Cole et al., 1992), on average, throughout the week (Sadeh et al., 2003).

#### Impulsive and psychopathic trait measures

##### BIS-11

The Barratt Impulsiveness Scale (BIS-11), a 30 item self-report questionnaire validated in impulsive and typical or control populations (Swann et al., 2001; Stanford et al., 2009), measured attentional impulsivity, motor impulsivity, and non-planning impulsivity. A total score of 74 or greater is considered high impulsivity. Cronbach's alpha for all 30 items of the scale was 0.82, suggesting the scores used in the current study are highly reliable.

##### TriPM

The Triarchic Psychopathy Measure (TriPM), a 58-item self-report questionnaire validated with adolescents and adults (age 14 and older), measures three specific constructs of psychopathy (boldness, meanness, and disinhibition) (Patrick, 2010). Higher scores indicate more psychopathic behaviors and traits, and when these scores are used together they predict the construct of psychopathy well as compared to other self-report measures (Patrick, 2010). Cronbach's alpha for all 58 items of the scale was 0.86, suggesting the scores are highly reliable.

#### Personality trait measures

##### PAI subscales

Broader aspects of negative affectivity were measured in 100 participants (58 women, 42 men) using the antisocial (i.e., 25 items; α = 0.84), anxiety (i.e., 23 items; α = 0.91), borderline (i.e., 24 items; α = 0.86), and depression (i.e., 24 items; α = 0.89) subscales of the PAI (Morey, 1991). These PAI subscales were included in the research design to screen for potential personality influences on sleep, and were added to the study design after initial study implementation. The PAI was developed for use in community and clinical populations (Kurtz et al., 1993; Salekin et al., 2001; Diamond and Magaletta, 2006; Jacobo et al., 2007; DeShong and Kurtz, 2013), with demonstrated good reliability and validity (Harty et al., 2010). PAI scales have a mean score of 50*T*, and a standard deviation of 10*T*, and as such a score of 70*T*, or higher is considered clinically significant (Morey, 1991; Morey and Quigley, 2002). PAI subscale scores are stable over time (Morey, 1991), consistent with their measuring stable personality traits.

### Procedure

Participants were tested individually in two sessions, each lasting approximately 30 min in length. In the first session, participants provided written informed consent and then completed self-report questionnaires administered on a computer located in a quiet room using secure online survey software (Qualtrics©, Survey Software). Next, a preprogrammed actiwatch was placed on the participant's preferred wrist. Participants were instructed to wear the watch continuously for a period of 7 days and given instructions to keep the watch dry and secure to the wrist. A small marker button was pressed to record the onset of rest and awakening times. After 7 days, participants returned the actigraph to the lab and the data was extracted using the Respironics Actiware 5 ActiReader. Also, at this time, the subset of the participants (*n* = 100) completed the antisocial, borderline, anxiety, and depression subscales of the PAI.

## Results

Table 1 summarizes the means and *SD* for women and men. Average sleep durations were above 8 h for women and men. However, 42.75% of the sample were self-described “poor sleepers” (i.e., PSQI scores > 5) and 16.58 and 11.14% of the sample showed average sleep durations of less the 6.5 h and < 6 h, respectively. Better reports of sleep quality correlated with longer durations of sleep across the subsequent week in men (*r*= −0.17, *p* < 0.05), but not women (*r* = 0.03). The incidence of sleep medication (i.e., both over the counter and prescription sleep medication) use to aid in sleeping during the past month was 16.06% (*n* = 62) in the sample, with 9.95% using medication less than once a week, and only 3.37 and 3.11% using medication one to two times a week or three or more times a week, respectively. Women reported using medication (*n* = 45) more often than men (*n* = 17), of these 20 women and 5 men reported using medication at least one to two times each week to aid in sleeping [$X_{\left(1,N=386\right)}^{2}$ = 6.20, *p* = 0.013]. Preliminary analyses established that excluding these participants did not change the direction or significance of the results reported below.

**Table 1:** **Descriptive statistics for variables of interest by gender**.

**Variables**	**Male *M* (SD)**	**Female *M* (SD)**	**Effect sex (*d)***
Age	18.75 (1.22)	18.48 (.74)	0.27^**^
**SELF-REPORT MEASURES**			
PSQI	5.01 (2.20)	5.57 (2.77)	−0.22^*^
BIS-11 Total	62.10 (9.55)	60.61 (10.91)	0.15
TriPM Total	67.07 (13.34)	52.63 (12.43)	1.12^***^
PAI-ANX (*T*)	51.33 (8.46)	56.40 (11.85)	−0.49^*^
PAI-DEP (*T*)	51.74 (9.38)	53.05 (11.95)	−0.12
PAI-BOR (*T*)	56.69 (10.20)	56.36 (11.16)	0.03
PAI-ANT (*T*)	59.17 (10.33)	53.12 (10.20)	0.59^**^
**ACTIGRAPHY MEASURES**			
Sleep Efficiency (% Sleep)	90.06 (6.35)	90.28 (6.20)	−0.04
Sleep Duration (min)	483.51 (161.57)	502.69 (138.94)	−0.13

*N = 386 (PAI N = 100)*.

* *p < 0.05(two−tailed)*,

** *p < 0.01(two−tailed)*,

*** *p < 0.00 (two-tailed)*.

Table 2 summarizes the means, *SD*, and correlations for the variables of interest. Scores on the personality measures were within the normal range, with the exception of 8–9% of the sample of 100 participants scoring at or above 70*T* on the depression or anxiety measures, and 11–12%of the sample in the clinical range on the antisocial behaviors and borderline measures. As expected, compared to men, women reported significantly lower levels of psychopathic traits and antisocial behavior as measured by the TriPM total score, *F*_(1, 384)_ = 0.66, *p* < 0.00, and the PAI, *F*_(1, 98)_ = 0.27, *p* < 0.01 and higher levels of anxiety on the PAI, *F*_(1, 98)_ = 5.02, *p* < 0.05. Furthermore, men in the current study were slightly older than women, *F*_(1, 384)_ = 12.39, *p* < 0.05, and reported better subjective sleep quality than women as measured by the PSQI, *F*_(1, 384)_ = 7.32, *p* < 0.05.

**Table 2:** **Overall means, standard deviations, and correlations**.

**Variables**	**1**	**2**	**3**	**4**	**5**	**6**	**7**	**8**	**9**	**10**
1. Age	−									
2. Sleep Duration (min)	−0.05	−								
3. Sleep Efficiency (% Sleep)	−0.04	0.21^**^	−							
4. BIS Score	−0.03	−0.09	−0.09	−						
5. PSQI Score	0.08	−0.04	0.02	0.17^**^	−					
6. TriPM Score	0.02	0.00	−0.04	0.36^**^	0.04	−				
7. PAI Anxiety	0.03	−0.07	−0.03	0.21^*^	0.25^*^	−0.07	−			
8. PAI Depression	0.07	−0.01	−0.23^*^	0.28^**^	0.36^**^	0.10	0.67^**^	−		
9. PAI Borderline	0.00	−0.04	−0.11	0.43^**^	0.17	0.27^**^	0.64^**^	0.68^**^	−	
10. PAI Antisocial	−0.07	−0.07	−0.01	0.42^**^	0.02	0.70^**^	0.15	0.20^*^	0.48^**^	−
Mean	18.59	494.69	90.19	61.23	5.34	68.65	54.27	52.50	56.50	55.66
Standard deviation	0.98	148.90	6.27	10.38	2.56	14.65	10.81	10.91	10.71	10.63

** *Correlation is significant at the 0.01 level (2-tailed)*.

* *Correlation is significant at the 0.05 level (2-tailed). Variables 1–6 (N = 386), and variables 7–10 (N = 100) Means and Standards Deviations were derived from non-standardized variables. Means and standard deviations for unstandardized values*.

### Impulsivity and psychopathic personality traits

As impulsivity is a central construct of psychopathic personality traits we examined the unique variance of both self-reported impulsivity (BIS-11 scores) and psychopathic (TriPM total scores) traits, on actigraph measures of average sleep efficiency (i.e., percent sleep) using a simultaneous multiple regression with these scores as predictors. The overall regression used to predict sleep efficiency for both trait impulsivity (BIS-11) and trait psychopathy (Tri-PM total) was not significant. Furthermore, no main effects emerged from the models.

Analyses were also conducted to assess whether BIS-11 scores and TriPM total scores significantly predicted the actigraph measure of average sleep duration (i.e., sleep time in minutes) by conducting a simultaneous multiple regression. The overall regression used to predict average sleep duration was not significant. Furthermore, no main effects emerged from the model.

To examine the effect of self-reported impulsivity (BIS-11 scores) and psychopathic (TriPM total scores) traits in the sample of 386 participants on subjective sleep quality (PSQI scores), a simultaneous multiple regression was included in analyses. The overall regression used to predict subjective sleep quality was significant. Furthermore, BIS-11 scores were significant predictors of subjective sleep quality, and TriPM total scores were not. That is, after controlling for psychopathy trait scores, impulsivity scores accounted for 2.80% of the variance in subjective sleep quality, such that a one point increase in BIS-11 scores resulted in a 0.04 predicted increase in subjective sleep quality score, indicating poorer subjective sleep quality. Thus, as trait impulsivity increased, subjective sleep quality decreased. See Table 3 for combined output for Sleep Efficiency, Sleep Duration, and Subjective Sleep Quality.

**Table 3:** **Summary of Simultaneous Multiple Regressions with TriPM and BIS-11 Predicting Sleep Efficiency (% Sleep), Sleep Duration (min), and Subjective Sleep Quality**.

	**Sleep efficiency (% Sleep) (*n* = 386)^a^**			**Sleep duration (min) (*n* = 386)^b^**			**Subjective sleep quality (PSQI) (*n* = 386)^c^**		
	***B***	***SE***	**β**	***B***	***SE***	**β**	***B***	***SE***	**β**
BIS-11	−0.06	0.03	−0.09	−1.42	0.78	−0.10	0.04	0.01	0.18^*^
TriPM	−0.00	0.02	−0.01	0.40	0.55	0.04	−0.00	0.01	−0.03

a *R^2^ = 0.01, F_(2, 383)_ = 1.65, p > 0.05*.

b *R^2^ = 0.01, F_(2, 383)_ = 1.66, p > 0.05*.

c *R^2^ = 0.03, F_(2, 383)_ = 5.77, p = 0.003*.

* *p < 0.05*.

Finally, to test the bidirectional relationship between impulsive and psychopathic traits and sleep variables, simultaneous regressions were included with each score (i.e., BIS-11 score and TriPM score) as dependent variables and sleep variables as predictors (i.e., sleep duration and sleep efficiency). The overall regression used to predict impulsive traits (i.e., BIS-11 scores) was not significant. Furthermore, no main effects emerged from the model. Furthermore, the overall regression used to predict psychopathic traits (i.e., TriPM scores) traits was not significant. No main effects emerged from the model. Additionally, a simultaneous regression was used to predict subjective sleep quality (i.e., PSQI scores) with sleep efficiency and sleep duration as predictors. The overall model was not significant. No main effects emerged from the model. Table 4 contains combined results for the above analyses.

**Table 4:** **Summary of Simultaneous Multiple Regressions with Sleep Duration (min) and Sleep Efficiency (% Sleep) Predicting TriPM, BIS-11, and PSQI Scores**.

	**TriPM scores (*n* = 386)^a^**			**BIS-11 scores (*n* = 386)^b^**			**PSQI scores (*n* = 386)^c^**		
	***B***	***SE***	**β**	***B***	***SE***	**β**	***B***	***SE***	**β**
Sleep Duration	−0.01	0.00	−0.07	0.00	0.01	0.01	0.00	0.00	−0.05
Sleep Efficiency	−0.13	0.09	−0.08	−0.09	0.12	−0.04	0.01	0.02	0.03

a *R^2^ = 0.01, F_(2, 383)_ = 2.53, p > 0.05*.

b *R^2^ = 0.00, F_(2, 383)_ = 0.29, p > 0.05*.

c *R^2^ = 0.00, F_(2, 383)_ = 0.45, p > 0.05*.

### Personality traits

A simultaneous multiple regression was used to examine the effect of the PAI measures of personality traits (e.g., antisocial, anxiety, depression, and borderline scores) on average sleep efficiency (i.e., percent sleep). The overall regression used to predict average sleep efficacy approached significance. Furthermore, PAI depression score were a significant predictor of sleep efficiency. That is, after controlling for PAI borderline scores, PAI anxiety scores, and PAI antisocial scores, depression *T*-scores accounted for 5.86% of the variance in sleep efficiency, such that a one point increase in depression *T*-scores resulted in a 0.10 predicted decrease in sleep efficiency. Furthermore, results of a linear regression including only PAI depression scores predicting sleep efficiency was significant. PAI depression scores were a significant predictor of sleep efficiency, such that PAI depression scores accounted for a 5.11% of the variance in sleep efficiency, a one point increase in depression *T*-scores resulted in a 0.10 predicted decrease in sleep efficiency. Thus, in both models, depression scores were significant predictors of sleep efficiency (i.e., percent sleep), and as depression scores increased, sleep efficiency decreased.

To examine the effect of PAI subscales on average sleep duration (i.e., sleep time in minutes), a simultaneous multiple regression was conducted. The overall regression used to predict average sleep duration was not significant, and no main effects emerged from the model. Furthermore, no regression analyses yielded significant results for each personality trait entered into the model individually. That is, PAI antisocial scores, PAI anxiety scores, PAI depression scores, and PAI borderline score did not predict sleep duration in the sample.

Furthermore, to test the bidirectional relationship between personality traits and sleep variables, simultaneous regressions were included with each score (i.e., PAI anxiety, PAI depression, PAI borderline, and PAI antisocial scores) as dependent variables and sleep variables as predictors (i.e., sleep duration and sleep efficiency). The overall regression used to predict anxiety scores, borderline scores, and antisocial scores were not significant and no main effects emerged from the models. However, the overall regression used to predict depression scores approached significance. Furthermore, sleep efficiency (i.e., percent sleep) was a significant predictor of depression scores. Table 5 contains combined results for the above analyses.

**Table 5:** **Summary of simultaneous multiple regressions with sleep duration (min) and sleep efficiency (% sleep) predicting PAI anxiety, PAI depression, PAI borderline, and PAI antisocial scores**.

	**Anxiety scores (*n* = 100)^a^**			**Depression scores (*n* = 100)^b^**			**Borderline scores (*n* = 100)^c^**			**Antisocial scores (*n* = 100)^d^**		
	***B***	***SE***	**β**	***B***	***SE***	**β**	***B***	***SE***	**β**	***B***	***SE***	**β**
Sleep duration	0.00	0.01	−0.06	0.01	0.01	0.07	0.00	0.01	−0.01	−0.01	0.01	−0.08
Sleep efficiency	−0.05	0.39	−0.01	−0.91	0.38	−0.25^*^	−0.40	0.38	−0.11	0.12	0.38	0.03

a *R^2^ = 0.01, F_(2, 97)_ = 0.22, p > 0.05*.

b *R^2^ = 0.01, F_(2, 97)_ = 2.84, p = 0.063*.

c *R^2^ = 0.01, F_(2, 97)_ = 0.64, p > 0.05*.

d *R^2^ = 0.01, F_(2, 97)_ = 0.27, p > 0.05*.

* *p < 0.05*.

A similar analysis examined the effect of PAI subscales on subjective sleep quality (PSQI scores). The overall regression used to predict PSQI scores was significant. Furthermore, PAI depression scores were a significant predictor of subjective sleep quality. That is, after controlling for PAI borderline scores, PAI anxiety scores, and PAI antisocial scores, depression *T*-scores accounted for 8.47% of the variance in subjective sleep quality, such that a one point increase in depression *T*-scores resulted in a 0.10 predicted increase in PSQI scores (i.e., indicating poorer subjective sleep quality). See Table 6 for combined output for Sleep Efficiency, Sleep Duration, and Subjective Sleep Quality.

**Table 6:** **Summary of simultaneous multiple regressions with anxiety, depression, borderline, and antisocial *T*-scores predicting sleep efficiency (% sleep), sleep duration (min), and subjective sleep quality**.

	**Sleep efficiency (% Sleep) (*n* = 100)^a^**			**Sleep duration (min) (*n* = 100)^b^**			**Subjective sleep quality (PSQI) (*n* = 100)^c^**		
	***B***	***SE***	**β**	***B***	***SE***	**β**	***B***	***SE***	**β**
Anxiety (*T*)	0.06	0.04	0.23	−1.68	2.22	−0.11	0.01	0.03	0.06
Depression (*T*)	−0.10	0.04	−0.36^*^	1.10	2.29	0.07	0.10	0.04	0.43^*^
Borderline (*T*)	−0.01	0.05	−0.05	0.18	2.59	0.01	−0.04	0.04	−0.16
Antisocial (*T*)	0.02	0.03	0.07	−1.08	1.84	−0.07	0.00	0.03	0.03

a *R^2^ = 0.08, F_(2, 95)_ = 1.65, p = 0.088*.

b *R^2^ = 0.01, F_(2, 95)_ = 0.27, p > 0.05*.

c *R^2^ = 0.14, F_(2, 95)_ = 3.93, p = 0.005*.

* *p < 0.05*.

### Gender differences

As recommended by Dawson (2014), to analyze the effect of gender on impulsivity and psychopathic traits on sleep efficiency (i.e., percent sleep), analyses using moderated multiple regression with gender in each model as a main effect and in interaction terms with the TriPM and BIS-11 (e.g., BIS-11 scores (centered) × Gender interaction; TriPM scores (centered) × Gender interaction; etc…) were conducted. No significant interactions were found. Similar analyses and results were found for the bidirectional analyses (i.e., sleep efficiency (centered) × Gender interaction predicting TriPM scores, sleep efficiency (centered) × Gender interaction predicting BIS-11 Scores); no significant interactions were found.

To test the externalizing variables of interest (BIS-11 and Tri-PM total scores) for men and women for average sleep duration (i.e., sleep time in minutes) a moderated multiple regression with gender in each model as a main effect and in interaction terms with the TriPM and BIS-11 (e.g., BIS-11 scores (centered) × Gender interaction; TriPM scores (centered) × Gender interaction; etc…) were conducted. No significant interactions were found. Similar analyses and results were found for the bidirectional analyses (i.e., sleep duration (centered) × Gender interaction predicting TriPM scores, sleep duration (centered) × Gender interaction predicting BIS-11 Scores); no significant interactions were found.

To examine effects of impulsivity (BIS-11 scores) and psychopathy (TriPM total scores) traits on subjective sleep quality (PSQI scores) a moderated multiple regression with gender in each model as a main effect and in interaction terms with the TriPM and BIS-11 (e.g., BIS-11 scores (centered) × Gender interaction; TriPM scores (centered) × Gender interaction; etc…) were conducted. No significant interactions were found. However, there was a main effect of gender for both models (results are presented in Table 7) suggesting that gender is an important factor in subjective sleep quality.

**Table 7:** **Summary of moderated multiple regressions with gender, BIS-11 (Centered), and Gender × BIS-11(Centered) predicting subjective sleep quality**.

	**Subjective sleep quality (PSQI) (*n* = 386)^a^**				**Subjective sleep quality (PSQI) (*n* = 386)^b^**		
**Model 2**	***B***	***SE***	**β**	**Model 2**	***B***	***SE***	**β**
Gender	0.62	0.26	0.12^*^	Gender	0.83	0.30	0.16^*^
BIS-11 (centered)	0.05	0.04	0.19	TriPM (centered)	−0.00	0.03	−0.02
Gender × BIS-11 (centered)	−0.00	0.03	−0.01	Gender × TriPM (centered)	0.02	0.02	0.14

a *R^2^ = 0.04, F_(2, 382)_ = 5.73, p = 0.001*.

b *R^2^ = 0.02, F_(2, 382)_ = 3.13, p = 0.026*.

* *p < 0.05*.

Interaction analyses were conducted with the variables of interest (PAI depression *T* scores, PAI anxiety *T* scores, PAI antisocial *T* scores, and PAI borderline *T* scores) using moderated multiple regression with gender in each model as a main effect and in interaction terms with the depression, anxiety, borderline, and antisocial scores (e.g., depression scores (centered) × Gender interaction; anxiety scores (centered) × Gender interaction; etc…) were conducted. No significant interactions were found for anxiety, borderline, or antisocial scores. However, model 2 of the depression scores (centered) × Gender when predicting sleep efficiency (i.e., percent sleep) was significant and a marginally significant interaction was found. Results are presented in Table 8. Similar analyses were conducted for the bidirectional analyses (i.e., sleep efficiency (centered) × Gender interaction predicting PAI anxiety scores, sleep efficiency (centered) × Gender interaction predicting PAI depression scores, etc…); no significant interactions were found.

**Table 8:** **Summary of moderated multiple regressions with gender, depression (centered), and gender × depression (centered) predicting subjective sleep quality**.

**Model 2**	**Subjective sleep quality (PSQI) (*n* = 100)**		
	***B***	***SE***	**β**
Gender	−0.43	0.58	−0.07
Depression (centered)	−0.23	0.10	−0.85^*^
Gender × depression (centered)	0.10	0.06	0.65

*R^2^ = 0.09, F_(2, 96)_ = 3.04, p = 0.033*.

* *p < 0.05*.

Interaction analyses were conducted with the variables of interest (PAI depression *T* scores, PAI anxiety *T* scores, PAI antisocial *T* scores, and PAI borderline *T* scores) using moderated multiple regression with gender in each model as a main effect and in interaction terms with the depression, anxiety, borderline, and antisocial scores (e.g., depression scores (centered) × Gender interaction; anxiety scores (centered) × Gender interaction; etc…) were conducted. No significant interactions were found. Similar analyses were conducted for the bidirectional analyses [i.e., sleep time (centered) × Gender interaction predicting PAI anxiety scores, sleep duration (centered) × Gender interaction predicting PAI depression scores, etc…]; no significant interactions were found.

To examine effects of personality traits (PAI anxiety, PAI depression, PAI borderline, and PAI antisocial scores) on subjective sleep quality (PSQI scores) with gender as a moderator analyses using moderated multiple regression with gender in each model as a main effect and in interaction terms with the depression, anxiety, borderline, and antisocial scores (e.g., depression scores (centered) × Gender interaction; anxiety scores (centered) × Gender interaction; etc…) were conducted. No significant interactions were found, however, there was a main effect of gender in all models (see Table 9 for regression table). Results suggest that women report worse subjective sleep quality than men.

**Table 9:** **Summary of moderated multiple regressions with gender, personality traits (centered), and gender × personality traits (centered) predicting subjective sleep quality**.

**Model 2**	**Subjective sleep quality (PSQI) (*n* = 100)^a^ Anxiety (IV)**			**Subjective sleep quality (PSQI) (*n* = 100)^b^ Depression (IV)**			**Subjective sleep quality (PSQI) (*n* = 100)^c^ Borderline (IV)**			**Subjective Sleep Quality (PSQI) (*n* = 100)^d^ Antisocial (IV)**		
	***B***	***SE***	**β**	***B***	***SE***	**β**	***B***	***SE***	**β**	***B***	***SE***	**β**
Gender	1.83	0.52	0.34	1.90	0.48	0.35	2.03	0.50	0.38	2.18	0.53	0.41
IV (centered)	0.02	0.10	0.06^*^	0.10	0.08	0.41^*^	0.20	0.08	0.42	−0.03	0.08	−0.12
Gender × IV (Centered)	0.02	0.05	0.11	−0.01	0.05	−0.07	−0.04	0.05	−0.25	0.04	0.05	0.26

a *R^2^ = 0.17, F_(2, 96)_ = 6.44, p = 0.001*.

b *R^2^ = 0.26, F_(2, 96)_ = 10.94, p = 0.000*.

c *R^2^ = 0.18, F_(2, 96)_ = 6.83, p = 0.000*.

d *R^2^ = 0.16, F_(2, 96)_ = 6.20, p = 0.001*.

* *p < 0.05*.

## Discussion

The aim of the current study was to examine the nature of the association between sleep and traits of both externalizing (i.e., impulsivity and psychopathy) and internalizing (i.e., anxiety and depression) traits in emerging adults. Sleep in a large number of young women and men (*N* = 386) was measured using actigraphy, a method that provides valid measures of sleep duration (i.e., sleep time in minutes) and sleep efficiency (i.e., percent sleep) (Tryon, 2004). Consistent with previous research using self-reports of sleep durations and sleep quality in this population (Tsai and Li, 2004; Maslowsky and Ozer, 2014), the majority of women and men had similar, adequate levels of sleep and men compared to women reported better subjective sleep quality and shorter sleep durations. As expected, scores on well-established measures of traits associated with externalizing personality disorders (i.e., BIS-11 total scores, TriPM total scores, and PAI subscales) were generally well below the clinical range. Also consistent with previous research (Blonigen et al., 2005; Wasserman et al., 2005), compared to men, women reported lower levels of psychopathic traits, antisocial behavior and higher levels of anxiety and depression. Thus, sleep and personality characteristics of our sample support the generalizability of our results.

We found that emerging adults reporting higher levels of impulsivity also reported poorer sleep quality on the PSQI. Associations between impulsivity and PSQI scores have been previously observed in research in forensic psychiatric settings (Kamphuis et al., 2013, 2014) and in individuals with clinical diagnoses such as ADHD (Mahajan et al., 2009). To our knowledge, this is the first report showing that stronger impulsive personality traits predict higher PSQI scores (i.e., poorer subjective sleep quality) in a community sample. Previous studies in American samples have investigated risk-taking behaviors (e.g., alcohol consumption using the Rutgers Alcohol Problem Index) and subjective sleep quality in university samples and found again that risk-taking behaviors increase as subjective sleep quality decreases (Kenney et al., 2012). Our results show these findings at the total score levels of well-established measures of the construct of impulsivity and add to previous research by using regression analyses in our data interpretation. Additionally, previous studies of undergraduate college students have shown that the thought processes related to impulsivity (e.g., urgency and counterfactual thoughts) cause sleep disturbances (i.e., assessed with the UPPS Impulsive Behavior Scale, the Bedtime Counterfactual Processing Questionnaire, and the Insomnia Severity Index) (Schmidt and Van der Linden, 2009), supporting our results that subjective sleep quality is affected by impulsive personality traits. Further, the small number of women in the forensic samples examined in the previous research precluded a meaningful test of sex differences. Therefore, it is notable that although men in this investigation generally reported more impulsivity than did women and women generally reported poorer sleep quality than did men, the relationship between the two variables was similar in both groups. Others have established in experimental paradigms that sleep disruption during adolescence and early adulthood increases impulsivity (Rossa et al., 2014). Our data suggest that even within the normal range of behavior, higher impulsivity in emerging adulthood is associated with more sleep complaints in both women and men.

It may be informative that we found no evidence of a similar relationship between impulsivity and actigraphy measures of sleep duration and sleep efficiency. It may be that the inverse relationship between quantitative aspects of sleep and impulsivity observed in clinical populations (Lindberg et al., 2003; Ireland and Culpin, 2006; Boonstra et al., 2007) does not exist in young women and men with adequate sleep and subclinical levels of externalizing personality traits. It may also be relevant that one explanation for low correlations between subjective sleep quality and actigraph measures is that changes in sleep architecture that influence subjective sleep quality may not influence sleep duration or efficiency (Lemola et al., 2013). Altered sleep architecture (i.e., higher levels of SWS) in men and women with APD who report poorer sleep quality and satisfaction (Lindberg et al., 2009) has been interpreted as evidence of altered brain development and abnormalities in the prefrontal cortex proposed to contribute to antisocial behavior. Therefore, actigraphy may be unrelated to impulsivity in this research because actigraphy is relatively insensitive to neural systems influencing both sleep architecture and externalizing behavior. Of course, it may be that a relationship between these variables would be observed in future studies using laboratory measures of impulsive and aggressive behavior and controlling for factors influencing sleep duration and efficiency, such as menstrual cycle phase (Tworoger et al., 2005).

It is also informative that our study showed no correlation between objective and subjective measures of sleep satisfaction and efficiency. These findings are consistent with previous studies in adolescence and emerging adulthood finding low to no correlation between these variables (Palermo et al., 2007; Lauderdale et al., 2008; Lang et al., 2013). A large study of adolescents and emerging adults (*N* = 1581; age range = 16–25 years) found no to weak relationships between subjective sleep quality [i.e., PSQI and Insomnia Severity Index (ISI) scores] and objective sleep quality (i.e., EEG assessment) (Lang et al., 2013). It may be that subjective measures of sleep satisfaction do not accurately reflect their actual sleep efficiency, particularly due to subjective overestimation of sleep duration (Lauderdale et al., 2008) and symptoms of depression or negative affectivity (Palermo et al., 2007). The results of the current study and previous studies continue to support the use of multiple assessment methods in sleep research.

A second aim of this research was to examine, for the first time in a community sample, the relationship between sleep and traits of psychopathy. Contrary to our findings for impulsivity, there was no support for a linear relationship between actigraph measures or subjective sleep and levels of psychopathic traits as measured by the Tri-PM or the antisocial subscale of the PAI. Although some researchers have found associations between self-reports of modest sleep deficiencies and increased reports of externalizing personality traits (Killgore et al., 2013), other research using self-report measures of sleep quality and behavior (Wolfson and Carskadon, 1998; Clinkinbeard et al., 2011; Meldrum and Restivo, 2014; Barnes and Meldrum, 2015) suggests marked deficiencies in sleep duration (e.g., < 5–6 h) may be required for the expression of antisocial behavior (i.e., delinquent and conduct problems). However, few individuals in this research (*n* = 14) showed average weekly sleep durations of < 5 h. Thus, whether this association represents a stable pattern of maladaptive behavior or transient effects of sleep deprivation on antisocial behavior requires further investigation in longitudinal research using a larger number of individuals with marked sleep deficiencies. This possibility, coupled with evidence that sleep disruption increases impulsivity, suggests it may be fruitful in future experimental research to examine the interaction between high and low levels of trait psychopathy and sleep disruption on the expression of impulsive behavior.

Finally, we also found that higher scores on the depression subscale of the PAI were associated with poorer subjective sleep quality and sleep efficiency across the prior 7 days of assessment. This finding that sleep was predicted by subsequent negative moods in young adults is consistent with other evidence of a bidirectional relationship between feelings of stress, depression, anxiety, and a decrease in percent sleep (Fuligni and Hardway, 2006; Doane and Thurston, 2014). In fact, our results further show that young adults with higher sleep efficiency (i.e., restful sleep) report lower levels of depression and negative affectivity. Adolescents with poorer sleep quality experience increased levels of negative affectivity, and those with higher scores on measures of negative affectivity show even poorer sleep quality (van Zundert et al., 2015). Studies on the bidirectional relationship between sleep and mood disorders in children and adolescents have shown that decreases in sleep also decrease positive affectivity and increase anxiety and depression (Babson et al., 2010; Talbot et al., 2010; Cousins et al., 2011). Other bidirectional analyses included in the study did not show significance but an association between mood and sleep variables is well-established (Baum et al., 2014). However, as scores on the PAI subscales in the present investigation fell well below the clinical range and less than half of our population rated themselves as poor sleepers, our results suggest a relationship between sleep and these aspects of negative affectivity extends to the subclinical or normal range of these behaviors in emerging adulthood. It may be that sleep complaints and lower sleep efficiency in individuals reporting higher levels of depression in this research is a marker of their increased risk for the development of internalizing disorders. Interestingly, whereas a similar relationship between subjective sleep quality and depressed mood was observed in both women men, our within sex analyses suggested an effect of depressed mood on sleep efficiency is more pronounced in young men.

Whether chronic sleep disruption in adolescence increases risk for externalizing personality disorders in adulthood remains a question for future longitudinal research and is a limitation in the current study. Additionally, future research may benefit from testing whether restriction of sleep duration for fewer than 5 h a week extends previous findings of increased externalizing traits in a larger sample of individuals with impaired sleep using objective measures (Wolfson and Carskadon, 1998; Meldrum and Restivo, 2014; Barnes and Meldrum, 2015). It will be useful for future longitudinal research to address this gap in the research. Using a community sample in the current study may represent a limitation as clinically significant scores were rare. Our cross-sectional design in a large typical (i.e., non-clinical) population of undergraduates supports the continuation of this research but limits the generalizability to emerging adults with clinically significant internalizing and externalizing traits. Furthermore, our study did not use repeated measures and as such directionality or causation cannot be determined. However, our findings are consistent with increasing research suggesting a bidirectional relationship between personality traits, particularly depression, and sleep (Cousins et al., 2011; Doane and Thurston, 2014) and suggest that both directions of causality are important, even at subclinical levels. The current study may have been limited by the overrepresentation of Caucasian participants (64.5%), however, our large sample is a strength of the study and ethnicity was not a main focus of the research design.

Use of the Pittsburgh Sleep Quality Index may have been a limitation in the current study due to low alpha scores; however, the reliability coefficients in the study were similar to meta-analytic findings of studies examining non-clinical populations (Mollayeva et al., 2015). Future studies of community and clinical samples may examine the difference between these populations on this measure. A final limitation of the study may be our inclusion of the TriPM, a triarchic measure of psychopathic traits that has received some criticism to date. The TriPM has been shown to be a reliable and valid measure of psychopathy in community samples (Drislane et al., 2014; Somma et al., 2015; Wall et al., 2015), but there continues to be a debate within the personality disorder research field regarding whether boldness is a significant feature of psychopathy (Miller and Lynam, 2012; Drislane et al., 2014). Some studies have found that boldness is a significant predictor of psychopathic rather than antisocial personality traits (Venables et al., 2014). Nevertheless, the debate of the triarchic model of psychopathy continues and may be a limitation to the current study. Thus, it is important that future research shed light on this debate and the operationalization of psychopathy and psychopathic traits.

In sum, our data add to increasing evidence of a relationship between sleep and personality traits associated with externalizing (i.e., oppositional and conduct) and internalizing disorders (i.e., depression). Longitudinal data suggests that both internalizing and externalizing disorders predict and are predicted by sleep difficulties (Shanahan et al., 2014). Therefore, the observed association between sleep and personality traits in young women and men not presenting with these disorders is consistent with a dimensional view of psychopathology (Cuthbert, 2005; Krueger et al., 2005) and so may inform an understanding of the development of psychological disorders.

## Author contributions

AY drafted the manuscript. All coauthors contributed to data analyses, interpretation of results, and revision of manuscript for intellectual content. The final version of the manuscript was approved by all coauthors. AY agreed to be accountable for all aspects of the work in ensuring that questions related to the accuracy or integrity of any part of the work are appropriately investigated and resolved.

### Conflict of interest statement

The authors declare that the research was conducted in the absence of any commercial or financial relationships that could be construed as a potential conflict of interest.
